# Antihypertensive, antidyslipidemic, and renoprotective effects of* Bursera simaruba* on metabolic syndrome

**DOI:** 10.22038/IJBMS.2023.66530.14601

**Published:** 2023-04

**Authors:** Elizabeth Alejandrina Guzmán Hernández, David Segura Cobos, María del Rosario González Valle, José del Carmen Benítez Flores, Rubén San Miguel Chávez, Leonardo del Valle Mondragón, Gil Alfonso Magos Guerrero, Pedro López Sánchez

**Affiliations:** 1 Medical Surgeon Career, Faculty of Superior Studies Iztacala, National Autonomous University of Mexico, Tlalnepantla, State of Mexico,54090, Mexico; 2 Histology Laboratory, Morphology and Function Unit, Faculty of Superior Studies Iztacala, National Autonomous University of Mexico, Tlalnepantla, State of Mexico, 54090, Mexico; 3 Phytochemistry Area, Postgraduate Degree in Botany, Campus Montecillo, Postgraduate College, Km. 36.5 México-Texcoco Road, Montecillo, Texcoco, State of Mexico, C.P. 56230, Mexico; 4 Department of Pharmacology, National Institute of Cardiology Ignacio Chávez, Mexico City, C.P. 04510, Mexico; 5 Department of Pharmacology, Faculty of Medicine, National Autonomous University of Mexico, Coyoacán, Mexico City, C.P. 04510, Mexico; 6 Postgraduate Studies and Research Section, Higher School of Medicine, National Polytechnic Institute, Mexico City, 11340, Mexico

**Keywords:** Blood pressure, *Bursera simaruba*, Hyperlipidemia, Kidney failure, Metabolic syndrome, Obesity, Oxidative stress

## Abstract

**Objective(s):**

Metabolic syndrome is associated with the development of chronic kidney disease. *Bursera simaruba* “chaca” is a medicinal plant used in Mexico for hypertension and empirical therapy. In this study, were examined the effects of ethanol extract of *B. simaruba* on metabolic syndrome.

**Materials and Methods:**

For induction of metabolic syndrome, 20% fructose was used, and it was administered in the water and food to male Wistar rats for 12 weeks, after administering ethanol extract of *B. simaruba* intragastrically (100 and 200 mg/kg/day) for 6 weeks, blood pressure was determined. In plasma, glucose, cholesterol, triglycerides, angiotensin II, oxide nitric, and angiotensin 1-7 were quantified. In the kidney was performed histological study and the activity of anti-oxidant enzymes was quantified.

**Results:**

Rats with metabolic syndrome developed obesity, arterial hypertension, dyslipidemia, and kidney damage characterized by proliferative glomerulonephritis, necrosis, and reduced activity of anti-oxidant enzymes. These alterations were significantly ameliorated by ethanol extract of *B. simaruba*.

**Conclusion:**

The ethanolic extract of *B. simaruba* showed antidyslipidemic, antihypertensive, anti-oxidant, and renoprotective effects.

## Introduction

Metabolic syndrome (MS) represents one of the main risk factors for the development of cardiovascular disease and diabetes mellitus; in Mexico, obesity represents one of the main public health problems, derived from changes in lifestyles, characterized by the consumption of foods rich in carbohydrates and fats, combined with a sedentary lifestyle. Despite the existence of effective drugs for the treatment of MS, their use is still limited, especially in rural areas, in Mexico the use of medicinal plants to treat various conditions continues to prevail (1-3). 

*Bursera* *simaruba* is a tree of the family *Burseraceae*, it produces lignans, and is widely used by the Mexican native population for different health issues; traditionally *B. simaruba* is used for its medicinal properties including relief from pain, inflammation, and rheumatism, and can help treat illnesses such as colds, skin tumors, polyps, venereal diseases, and hypertension (4, 5). 

The objective of this study was to evaluate the effect of ethanol extract of *B. simaruba *in MS. 

## Materials and Methods

*B. simaruba* was collected in the municipality of Cerro Azul, Veracruz, Mexico (N21°11″, W97°44′00″), It was identified by botanists at the National Autonomous University of Mexico. 

For *in vivo* evaluation, 3 kg of fresh leaves of *B. simaruba *were obtained, they were allowed to dry, later they were macerated in ethanol for 14 days, and an exhaustive extraction was carried out at reduced pressure using a rotary evaporator to eliminate the solvent until a volume of 50 ml was obtained, and dried in an oven at 40 °C.

***Determination of phenolic acids, flavonoids, and terpenoids***

The HPLC analysis was carried out in the Phytochemistry Laboratory of the Postgraduate College under the supervision of Dr Marcos Soto Hernández and with the advice of M. en C. Rubén San Miguel Chávez. The determination of phenolic acids, flavonoids, and terpenoids present in the ethanolic extract of *B. simaruba* was carried out in a Hewlett Packard® 1100 series liquid chromatograph, equipped with an automatic injector (Agilent®, 1200 Series Mod. G1329A) and a Hewlett Packard® 1100 series diode array detector. 

***Induction of metabolic syndrome***

Male Wistar rats with an initial weight of 250 ± 15 g were used and were provided by the Bioterium of the Faculty of Higher Studies Iztacala, which was carried out under the Mexican Norm (NOM-062- ZOO-1999). For the induction of MS, following the methodology described by Merino *et al*., (2014), rats with MS were treated orally for 6 weeks, integrating the following groups: captopril (30 mg/kg) (MS + CAP) (7), atorvastatin (10 mg/kg) (MS+ ATOR) (8), and ethanolic extract of *B. simaruba* (100 and 200 mg/kg) (MS+ EtOH).

The biochemical analyses in plasma (total glucose, cholesterol, and triglyceride) were measured using an Accutrend Sensor glucometer (Roche); systolic arterial blood pressure (SBP) was measured noninvasively using a tail-cuff computer-aided monitoring device (Automatic Blood Pressure Computer, Model LE 5007; Letica Scientific Instruments, Barcelona, Spain) using the procedures described by Guzman *et al*. (2015); it was carried out at the beginning (0 weeks), middle (12 weeks), and end (18 weeks).

The animals were placed in individual metabolic cages to determine the consumption of water, food, and urinary volume. Urinary protein was measured using the Bradford method (9). Rats were later sacrificed, and blood was collected (3 ml) from the abdominal vein for biochemical analysis of high-density lipoprotein cholesterol (HDLc) (Spinreact, Cat. 1001097) and LDLc (Spinreact, Cat. 41023) levels were measured using commercially available kits following the manufacturer´s protocol. The cardiac and atherogenic index was determined according to what was described by Ajiboye *et al*. (2014).

For the determination of plasma concentration of angiotensin II, angiotensin (1-7), nitric oxide, and endothelin were measured using Capillary Electrophoresis (Tenorio *et al*., 2010).

Kidney cortex tissue samples were obtained for histological analysis and anti-oxidant activity of catalase and superoxide dismutase enzymes, as described by Ajiboye *et al*. (2014). 

***Statistical analysis***

The data are the mean ± SEM, statistical analyses were performed using GraphPad Software (USA), and their interactions by two-factor analysis of variance and means were compared using Tukey´s multiple comparisons *post hoc* test.

## Results

**Figure 1:**
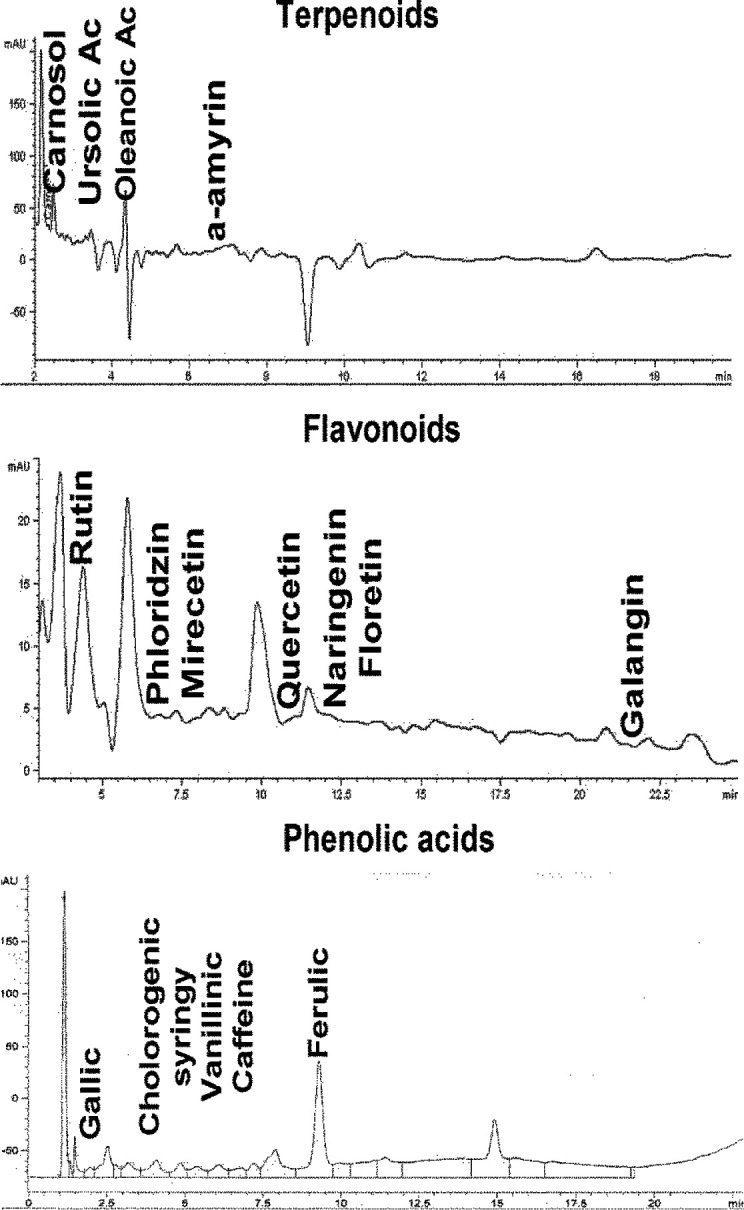
Chromatographic profile of ethanolic extract of Bursera *simaruba*

The analysis of high-performance liquid showed the presence of terpenoids: α-amyrin (35%), oleanolic acid (22.8%), and ursolic acid (9.71%); Flavonoids: naringenin (6.7%), phloridzin (2.28%); Phenolic acids: ferulic acid (5.15%), chlorogenic acid (2.9%), caffeic (0.5%), ferulic (5.15%), gallic acid (0.11%), and syringic acid (0.22%) (Figure 1 and Table 1).

***Effect of ethanolic extract of B. simaruba on metabolic syndrome***

In the present study we show that after the administration of 20% fructose in the water and food for 12 weeks, the animals developed three of the 5 risk factors characteristic of MS: obesity, quantified through body mass index, abdominal circumference, and Lee’s index; dyslipidemia; and arterial hypertension (Table 2). As can be seen in Table 3, captopril, atorvastatin, and ethanolic extract of *B. simaruba *with the dose of 100 and 200 mg/kg decreased the three indicators of obesity, which suggests that ethanolic extract from *B. simaruba* has an anti-obesity effect. 

**Figure 2:**
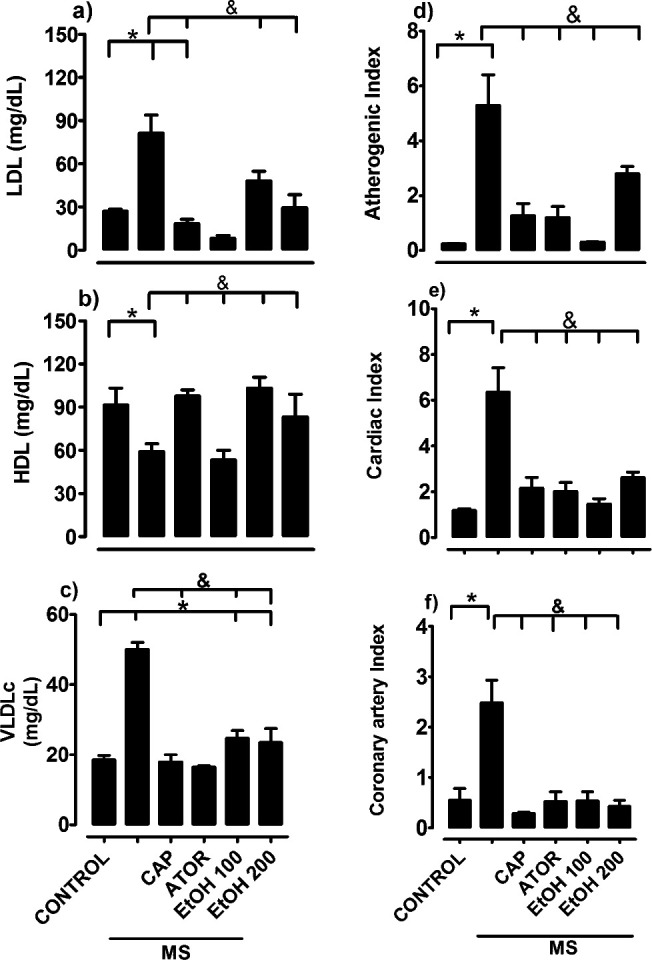
Lipid profile: (a) LDL, (b) HDL, (c) VLDL, (d) atherogenic, (e) cardiac, and (f) coronary artery indexes in rat. Values are the mean ± SEM (n = 6), *P<0.05 control vs treatment; &*P*<0.05 metabolic syndrome (MS) vs treatment. captopril (CAP), atorvastatin (ATOR), ethanolic extract (EtOH)

The dyslipidemia was characterized by an increase in the total concentration of triglycerides, low-density lipoprotein, and very low-density lipoprotein (Figure 2a); treatment with ethanolic extract of *B. simaruba,* with the dose of 100 and 200 mg/kg, decreased the plasmatic concentration of total lipids and increased high-density lipoprotein (Figure 2b), which suggests a protective effect of cardiovascular and arteriogenic risks (Figures 2 d, 2e, and 2f).

***Effect of ethanolic extract B. simaruba on hypertension***

**Figure 3:**
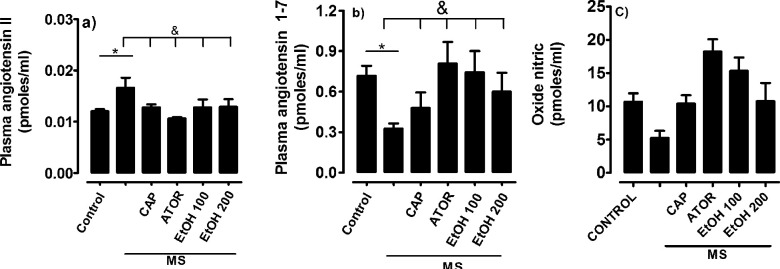
Effect of ethanol extract of Bursera *simaruba* on the plasma level of (a) nitric oxide, (b) angiotensin II, and (c) angiotensin 1-7. All values are represented as mean ± SEM. n = 6; *P<0.05 control vs treatment; &*P*<0.05 metabolic syndrome (MS) vs treatment. captopril (CAP), atorvastatin (ATOR), ethanolic extract (EtOH)

**Figure 4:**
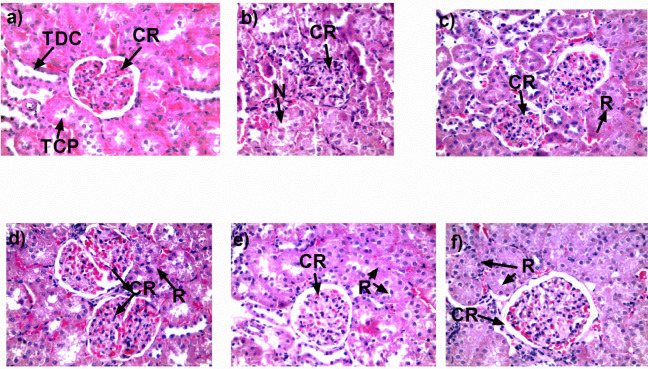
Photomicrographs in which different regions of the renal cortex. (CR) renal tissue with degenerative and necrotic change (N). (R) regenerated renal tissue. a) control, b) metabolic syndrome (MS), c) MS + captopril, d) MS + atorvastatin, e) MS + ethanolic extract 100 mg/kg, and f) MS + ethanolic extract 200 mg/kg

To determine if the ethanolic extract of *B. simaruba* influences arterial hypertension, blood pressure was determined after six weeks of treatment as can be seen in Figure 3; animals with MS increased blood pressure (MS: 148 ± 3 mmHg vs 100 ± 5 mmHg control) (Figure 3). This increase was associated with an increase in plasma Ang II and a decrease in vasodilator mediators such as nitric oxide and angiotensin 1-7 (Figure 4b). EtOH showed a preventive effect on the increase in blood pressure induced after MS and was associated with increased nitric oxide and plasma angiotensin 1-7 (Figures 4a and 4c).

***Effect of ethanolic extract B. simaruba on damaged kidney***

**Figure 5:**
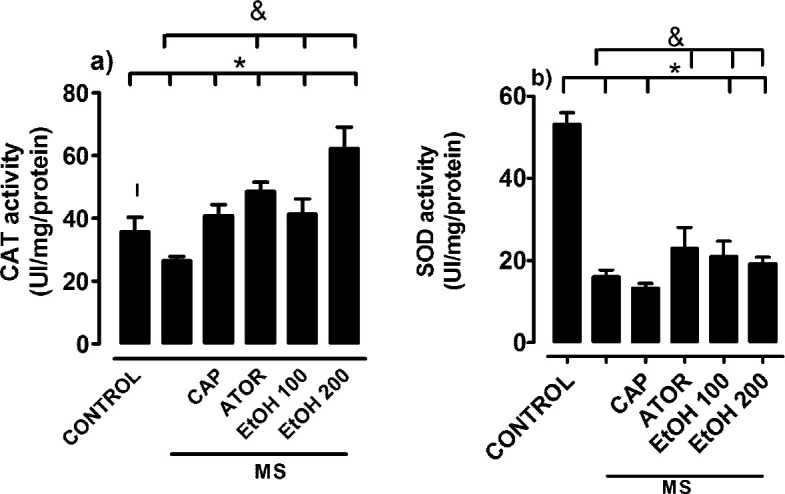
Effects of EtOH of Bursera *simaruba* on anti-oxidant enzymes, (a) catalase (CAT) and (b) superoxide dismutase (SOD). All values are represented as mean ± SEM. n = 6; *P<0.05 control vs treatment; &*P*<0.05 Metabolic syndrome (MS) vs treatment. captopril (CAP), atorvastatin (ATOR), ethanolic extract (EtOH)

In this study it was found that animals with MS developed kidney damage characterized by hypertrophy that was determined by kidney weight/total body weight ratio, (Control: 2.0 ± 0.02, MS: 2.5 ± 0.02 g); urine protein excretion (MS: 120 ± 5.3 mg/24 hr vs 20.6 ± 2.6 mg/24 hr control), and oxidative stress characterized by a decrease in the activity of anti-oxidant enzymes catalase and superoxide dismutase (Figures 5a and 5b). Histologically it was observed as proliferative glomerulonephritis, mainly affecting the mesangial cells. The inflammatory phenomenon extends into the interstitium, in which the presence of protein deposits was observed. The most affected glomerular structure corresponds to the proximal contoured tubules (TCP), followed by distal contoured tubules (TCD) (Figure 4b), in which the presence of moderate and multifocal necrosis was observed. The ethanolic extract of *B. simaruba* (100 and 200 mg/kg) reversed renal hypertrophy (EtOH 100 and 200 mg/kg):2.0 ± 0.035 and 1.98 ± 0.025 g), decreased excretion of proteins and activity of anti-oxidant enzymes were restored (Figures 5a and 5b). Histologically it was observed that necrosis was reduced, mainly in the proximal contoured tubules, and the regeneration of the renal corpuscle and the proximal contoured tubules was promoted, mainly, regenerative phenomenon occurs simultaneously by regions; in renal corpuscle, glomerular capillaries have narrow light and space of small Bowman’s capsule, indicating poor circulation and absence of glomerular filtration (Figures 4e and 4f). 

In the areas with regeneration, a little interstitium was observed, so there are no blood vessels and the proximal contoured tubules consist of cords of epithelial cells without a lumen or with very narrow light. The epithelial cells of these cords can be cubic and have no developed microvilli edges. Occasionally, cells in mitosis and nucleated cells can be identified (Figures 4e and 4f). 

Regarding treatment with captopril, similar effects were found although the regeneration is lower compared with the ethanolic extract of *B. simaruba*. Atorvastatin treatment reduces tubular necrosis (Figure 4c). 

**Table 1:** Phytochemical profile of ethanolic extract of Bursera *simaruba*

Retention time (min)	Area (mAU*s)	Variables	%
11.975	230.2104	Naringenin	6.7
22.122	230.3120	Galangin	0.83
4.382	645.0718	Routine	1.2
6.755	171.1739	Phloridzin	2.28
7.308	184.5394	Mirecetin	1.14
11.064	164.5262	Quercetin	0.47
12.780	200.610	Floretin	1.69
6.433	989.6905	-amyrin	35.8
4.367	884.8963	Oleanolic acid	22.85
2.481	501.307	Ursolic acid	9.71
2.335	93.7418	Carnosol	0.067
9.287	232.094	Ferulic acid	5.15
4.087	539.6374	Chlorogenic acid	2.9
1.952	158.8592	Gallic acid	0.11
4.854	310.8480	Syringic acid	0.22
5.378	335.7277	Vanillinic acid	0.43
6.092	371.7733	P-hydroxy benzoic	0.33
6.799	276.225	Caffeic	0.5
9.937	390.402	P-cumaric acid	0.26

**Table 2:** Indicators of metabolic syndrome in rat

**Variables**	**Control**	**MS**
**Initial body weight (g)**	240 ± 8	256 ± 10
		
**Body weight at 12 weeks (g)**	416 ± 6	520 ± 9*
**Lee index**	0.28 ± 0.01	0.38 ± 0.02*
**Plasma glucose (mg/dl)**	72 ± 5	104 ± 4*
**Plasma triglycerides (mg/dl)**	80 ± 3	248 ± 43*
**Plasma cholesterol (mg/dl)**	100 ± 0	100 ± 0
**Systolic blood pressure (mmHg)**	110 ± 10	160 ± 8*

**Table 3:** Effect of ethanolic extract (*Bursera simaruba*) on metabolic syndrome in rat

Variables	Control	MS	CAP	ATOR	EtOH 100	EtOH 200
Body weight at 18 weeks (g)	530 ± 17	616 ± 10*	568 ± 16&	568 ± 20&	576 ± 17&	562 ± 20&
Body mass index (g/cm^2^)	0.88 ± 0.02	1.4 ± 0.2*	0.86 ± 0.03&	1.1 ± 0.14&	1.23 ± 0.04&	1.18 ± 0.02&
Abdominal circumference (cm)	21 ± 0.37	24 ± 0.3*	18 ± 0.3&	22 ± 0.3&	19 ± 0.3&	20 ± 0.4&
Lee index	0.30 ± 0.01	0.36 ± 0.02*	0.31 ± 0.01&	0.33 ± 0.002	0.32 ± 0.03&	0.31 ± 0.03&
Plasma glucose (mg/dl)	80 ± 7	88 ± 4	84 ± 2	88 ± 3	87 ± 5	90 ± 4
Plasma triglycerides (mg/dl)	112 ± 16	220 ± 18*	185 ± 10&	166 ± 15&	145 ± 12&	100 ± 10&
Plasma Cholesterol (mg/dl)	100 ± 2	110 ± 15	95 ± 10	100 ± 2	100 ± 10	105 ± 10

Control, metabolic syndrome (MS), MS + captopril (CAP), MS + atorvastatin (ATOR), MS + EtOH 100 and MS + EtOH 200 mg/kg). n = 6; * *P*<0.05 control vs treatment, & *P*<0.05 metabolic syndrome vs treatment

## Discussion

MS is due to a group of risk factors and increases the risk of cardiovascular diseases and type 2 diabetes mellitus; its prevalence has increased in recent years worldwide, representing a public health problem. Lifestyle changes can reverse the components of MS, but sometimes pharmacological intervention is necessary for stricter control of these risk factors. We investigated the antihypertensive, antidyslipidemic, and renoprotective effects of the ethanolic extract of *B. simaruba* on the MS model in rats.

The present study showed that after 12 weeks of ingesting 20% fructose in water and food, three of the 5 risk factors that make up MS (arterial hypertension, obesity, and dyslipidemia) were consistent with the previous studies (6, 10, 12).

The accumulation of fat and high triglyceride levels observed in rats with MS in the present study explains the increase in body weight. Three indicators of obesity including the percentage of body weight gain, BMI, and abdominal circumference were measured; treatment with ethanolic extract showed antihyperlipidemic and anti-obesity effects; these effects may be due to the presence of caffeic acid, chlorogenic acid, α−amyrin, and naringenin in the extract; these secondary metabolites are inhibitors of fatty acid synthase and peroxisome proliferator-activated receptor alpha expression in the liver (13, 14). 

Free fatty acids can bind to specific receptors on endothelial cells, causing damage to the proper functioning of these cells, so they can be damaged and stop the synthesis of compounds such as nitric oxide and increase the effect of vasoconstrictor mediators such as angiotensin II. We found that after 12 weeks of induction of MS, the activity of the renin-angiotensin system increases, since treatment with captopril blood pressure is reduced by up to 60%, as has been shown in other works (15,16). The administration of the ethanolic extract of *B. simaruba* for 6 weeks showed a preventive effect on the increase in blood pressure induced by the MS, this effect can be attributed to the presence of caffeic and chlorogenic acids, that according to literature inhibit the activity of the angiotensin-converting enzyme (17, 18).

Excess free fatty acids can accumulate around kidneys, and this accumulation can cause lipotoxicity, loss of proper functioning, and adaptive changes such as hypertrophy and proteinuria (19, 20). 

The ethanolic extract of *B. simaruba* slowed the progression of functional and structural damage to the kidney in MS, this effect was attributed to the presence of oleanolic, ursolic, and ferulic acids in other studies. The presence of these secondary metabolites has shown a renoprotective effect through decreasing apoptosis in the kidney (21-23). 

Researchers demonstrated that high-fructose feeding increases oxidative stress resulting from an imbalance between the production of free radicals such as reactive oxygen species and reactive nitrogen species and the production of endogenous anti-oxidant defenses such as superoxide dismutase, catalase, and reduced glutathione; in MS rats they are decreased (23-25). The ethanolic extract of *B. simaruba *restored the activities of anti-oxidant enzymes, which suggests that part of the mechanism of action by which *B. simaruba* has a renoprotective effect is its anti-oxidant effect, as has been shown in other studies. Flavonoids have phenolic hydroxyl groups and excellent iron chelation properties and other transition metals, or through their binding to transcription factors, one of the compounds present in EtOH is oleanolic acid, which has been shown to activate the redox transcription factors as nuclear factor erythroid 2 (Nrf2) (26, 27). 

## Conclusion

The ethanolic extract of *B. simaruba* showed antidyslipidemic, antihypertensive, anti-oxidant, and renoprotective effects.

## Authors’ Contributions

DSC conceived the study. GAMG designed the study. DSC defined the intellectual content. DSC, MDR GV, LDC BF, R SMC, LDVM, GAMG, PLS, and EAGH performed the literature search. DSC, MDRGV, LDCBF, RSMC, LDVM, GAMG, PLS, and EAGH performed experimental studies. DSC, MDRGV, and LDCBF performed data acquisition.RSMC, LDVM, GAMG, PLS, and EAGH analyzed the data. GAMG, PLS, and EAGH performed statistical analysis. DSC, MDR GV, LDC BF, R SMC, LDV M, GA MG, PLS, and EAGH prepared the manuscript.

## Conflicts of Interest

The authors declare that no conflict of interest exists.
